# Human Periapical Odontogenic Granulomas: Aspects of Microvessel Density (MVD), Heterogeneity of Blood Vessels and Mast Cells Density (MCD)

**DOI:** 10.3390/biomedicines11102709

**Published:** 2023-10-05

**Authors:** Ciprian Roi, Meda Lavinia Negruțiu, Alexandra Roi, Mircea Riviș, Ruxandra Elena Luca, Marius Raica, Raluca Amalia Ceaușu, Alexandru Cătălin Motofelea, Pușa Nela Gaje

**Affiliations:** 1 Department of Anesthesiology and Oral Surgery, Multidisciplinary Center for Research, Evaluation, Diagnosis and Therapies in Oral Medicine, “Victor Babeș” University of Medicine and Pharmacy, Eftimie Murgu Sq. no. 2, 300041 Timisoara, Romania; ciprian.roi@umft.ro; 2 Department of Prostheses Technology and Dental Materials, Research Center in Dental Medicine Using Conventional and Alternative Technologies, Faculty of Dental Medicine, “Victor Babeș” University of Medicine and Pharmacy, 2 Eftimie Murgu Sq., 300041 Timisoara, Romania; negrutiu.meda@umft.ro; 3 Department of Oral Pathology, Multidisciplinary Center for Research, Evaluation, Diagnosis and Therapies in Oral Medicine, “Victor Babeș” University of Medicine and Pharmacy, Eftimie Murgu Sq. no. 2, 300041 Timisoara, Romania; alexandra.moga@umft.ro; 4 Department of Oral Rehabilitation and Dental Emergencies, The Interdisciplinary Center for Dental Medical Research, Lasers and Innovative Technologies, “Victor Babeș” University of Medicine and Pharmacy, 2 Eftimie Murgu Sq., 300041 Timisoara, Romania; luca.ruxandra@umft.ro; 5 Department of Microscopic Morphology/Histology, Angiogenesis Research Center, “Victor Babeș” University of Medicine and Pharmacy, 300041 Timisoara, Romania; marius.raica@umft.ro (M.R.); ra.ceausu@umft.ro (R.A.C.); gaje.nela@umft.ro (P.N.G.); 6 Department of Internal Medicine, Faculty of Medicine, “Victor Babeș” University of Medicine and Pharmacy, 300041 Timisoara, Romania; alexandru.motofelea@umft.ro

**Keywords:** MVD, mast cells, inflammation, tooth infection, periapical granuloma, CD34

## Abstract

Periapical odontogenic granulomas are among the most encountered pathology that involve the alveolar bone, with severe consequences such as bone resorption, the presence of inflammatory infiltrate and the formation of abnormal vascularization. The present study aimed to quantify the existence of the microvessel density (MVD), mast cell density (MCD) and heterogeneity of the encountered blood vessels. A total of 37 patients diagnosed with odontogenic periapical granulomas were included, and the gender distribution, age and localization of the pathological lesions was assessed. After the surgical removal of the periapical odontogenic granuloma, the collected tissue was fixed in 10% buffered formalin. Primary processing, morphological analysis and immunohistochemical staining was performed in order to characterize the altered tissue. The results outlined the presence of a high number of mast cells, especially in the area of the inflamed tissue; the high heterogeneity of the blood vessels; and increased MVD with positive CD34. The conclusions of the study focus on the key role of the mast cells and their implication in the initiation and development of the angiogenesis process, triggering the inflammatory response of the host. Nevertheless, periapical odontogenic granulomas develop as an inflammatory response to the interaction between the host’s immune system and microbial invasion.

## 1. Introduction

Edentations in the oral cavity, as a consequence of the loss of dental units, are mostly caused by damage to the supporting tissues, due to bacterial invasion from infected root canals emerging into the periapical space. Periapical lesions due to odontogenic causes are a common pathology affecting the maxillary bones, being one of the most frequent complications that eventually leads to the necessity of complex treatments. These lesions are a consequence of a bacterial infection initially localized in the root canals, which leads to the necrosis of the dental pulp, further evolving and developing apical periodontitis with the destruction of the periodontal ligament and the lysis of the alveolar bone [1,2]. 

Due to the inability of the host defense mechanisms to eradicate the infection, chronic periapical lesions are formed. Their origin is inflammatory, and basically, they represent the response of the periapical tissues to the microbial and chemical stimuli. This response involves the recruitment and activation of inflammatory cells, the generation of cytokines, the release of lytic enzymes and the activation of osteoclasts, which will result in alveolar bone resorption. In this case, the osteolysis of the jaw bones also determines the replacement of the bone tissue with granulation tissue, forming periapical granulomas [3,4].

Being a chronic inflammatory process involved into the appearance of granulation tissue, angiogenesis and lymphangiogenesis play a crucial role in the development and progression of periapical granulomas. The occurrence of angiogenesis in the pathologies of the different structures of the oral cavity is a negative prognostic factor that potentiates the manifestations of the disease and worsens its progression. The process is defined as the formation of new blood vessels from the ones that are already present in the area. It is a complex process that implies the migration and proliferation of endothelial cells and the formation and subsequent organization of tube-like structures that will eventually unify, resulting in a final network of stable blood vessels. Through this process, the inflamed and affected tissues are infused with pro-inflammatory cells and mediators, but also oxygen and nutrients brought by the neoformation vessels [3,5,6].

Inflammatory infiltrate and neo-angiogenesis influence the further development and growth of periapical granulomas. Inflammation and angiogenesis are actively involved in the progression of lesions, being codependent processes. During inflammation, angiogenic factors involved in angiogenesis are released by activating different types of cells: endothelial cells, mast cells, fibroblasts and macrophages. Chronic apical periodontitis is actually a type of immune defense response, involving multiple inflammatory cells and cytokines [7]. 

Mast cells are essential cells of the immune system and belong to the hematopoietic lineage. They are found in all types of connective tissue in the oral cavity, including the periodontal ligament, dental pulp (occasionally) and gingiva. The induction of inflammation by mast cells is a consequence of the release of preformed biological mediators as well as secondary mediators. Periapical lesions are a response of the tissues surrounding the tooth root to these mediators emerging from the dental pulp through the root canal system. Among the cells identified in periapical lesions, mast cells were detected in periapical granulomas and cysts, suggesting a crucial role of mast cells in the inflammatory mechanism of these lesions. Mast cells can subsequently synthesize and secrete additional mediators that are not preformed in their granules. Mediators present in mast cell granules can be grouped into two categories: preformed and de novo. Preformed mediators are represented by tryptase, chymase, cathepsin G, histamine, heparin, serotonin and TNF-α (tumor necrosis factor α). Mediators synthesized following mast cell activation are represented by interleukins IL-1, IL-3, IL-4, IL-5, IL-6, IL-8, IL-10, IL-13 and IL-16; activation factor of platelet factor (PAF); RANTES; MIF-1 alpha (macrophage inhibitory factor); and metabolites of arachidonic acid, prostaglandins and leukotriene C4 (LTC4). In addition to conventional mediators and cytokines, mast cells are able to secrete some growth factors, such as vascular endothelial growth factor (VEGF), fibroblast growth factor and nerve growth factor (NGF), which contribute to homeostasis or angiogenesis [8,9,10]. 

The present study aims to evaluate presence of the microvessel and mast cell density (MVD and MCD) in the odontogenic periapical granulomas and asses the heterogeneity of the blood vessels identified, focusing on their implications in the pathogenic process and evolution of the periapical granulomas.

## 2. Materials and Methods

The study was approved by the Ethics Committee of “Victor Babeș” University of Medicine and Pharmacy Timișoara (no. 39/2022), and patients agreed and signed an informed consent form that followed the guidelines of the Declaration of Helsinki. The study was conducted during September 2022–July 2023.

### 2.1. Patients’ Data

The following inclusion and exclusion criteria were applied:

Inclusion criteria:

- Age: 18–70 years;
- Both genders;
- Scheduled patients with the indication of tooth extractions;
- The presence of periapical granuloma identified on the Panoramic Dental X-ray.

Exclusion criteria:

- Age under 18 years;
- Patients diagnosed with neoplasms;
- Patients with acute diseases of the oral mucosa;
- Patients with altered general conditions: leukemia, recent myocardial infarction or stroke in the last 6 months;
- Patients undergoing drug treatment for bone pathologies (e.g., bisphosphonates).

After the application of the inclusion and exclusion criteria, a total of 37 patients were included in the study, 17 females and 20 males, with an age range from 24 to 72 years. Regarding the smoking status, 27 patients were non-smokers and 10 were smokers. The data are presented in Table 1.

### 2.2. Tooth Extraction

The tooth extraction was performed, followed by the curettage of the alveolar bone in order to remove and collect the periapical granuloma. Out of the included patients, 31 patients had 1 tooth extracted, 5 patients had 2 teeth extracted and 1 patient had the indication of extracting 3 teeth, as shown in Table 2.

Regarding the localization of the teeth with periapical granuloma that were extracted, 23 teeth were located on the upper maxilla and 14 on the mandible, as shown in Table 3.

The collected tissue of the periapical granulomas was further immersed in formalin, and the fixation of the specimens was carried out with 10% buffered formalin for 48–72 h.

### 2.3. Primary Processing

We performed an observational study on paraffin-embedded periapical tissue sections of 37 periapical granuloma specimens that were processed according to the standard histological technique.

After washing, they were dehydrated, clarified and then embedded in paraffin. For the inclusion step, we used the Thermo Shandon standardized inclusion automat (Thermo Fisher Scientific Inc., Arendalsvägen 16–418 78 Gothenburg, Sweden). The sectioning was carried out with the Shandom ME microtome. From each paraffin block, two sections, each approximately 3 to 5 μm thick, were cut. One set of sections was routinely stained with hematoxylin–eosin to confirm the clinical diagnosis, and the other set of sections was immunohistochemically stained to identify mast cells and blood vessels.

### 2.4. Morphologic Analysis

Morphological staining was carried out with the standard technique, using the automated staining machine (Leica Autostainer XL, Leica Biosystems Nussloch GmbH, Nußloch, Germany). The procedure used two staining solutions, Harris hematoxylin and eosin Y. Sections were deparaffinized and hydrated, then stained with Harris hematoxylin for 30 s. After washing, eosin staining followed for 2 min. After applying eosin, the parts were washed again, and then dehydrated and clarified in benzen.

### 2.5. Immunohistochemical Technique

Double CD34-mast cell tryptase immunostaining was used for the immunohistochemical evaluation of microvessel density (MVD) and mast cell density (MCD). The immunohistochemical detection of blood vessels was performed using mouse monoclonal antibodies with affinity for vascular endothelia (CD34, Mouse Monoclonal Anti-Human Endothelial Cell Marker, clone QBEnd/10). Mast cells were highlighted using mouse monoclonal antibodies with affinity for human mast cell tryptase (Mouse monoclonal anti-Human Mast cell tryptase, clone-10D11). 

The immunohistochemical method was performed through a fully automated and standardized procedure for all cases included in the study. The automatic Leica Bond-Max (Leica Biosystems, Newcastle upon Tyne, UK) was used for double immunostaining. Paraffin sections were treated with Bond Epitope Retrieval Solution 2 for 20 min (Leica Biosystems, Newcastle Ltd.). Endogenous peroxidase was blocked with 3% hydrogen peroxide solution for 5 min. The sections were then incubated for 30 min with the primary antibody—CD34 (Leica Bond, RTU, clone QBEnd/10). 

For visualization, we used the Bond Polymer Refine Detection System, which included the secondary antibody (8 min) and the polymer with an 8 min incubation time. After peroxidase blocking, the second anti-mast cell tryptase antibody (Leica Bond, RTU, clone 10D11) was applied. The Bond Polymer Refine Red detection system containing 3,3-diamino-benzidine dihydrochloride and hematoxylin was used for visualization. Stained sections were permanently mounted with Canada balsam.

### 2.6. Microscopic Evaluation and Image Analysis

Morphologically and immunohistochemically stained sections were analyzed using a Zeiss Axiocam 506 microscope (Jena, Germany), equipped with a real-time imaging system and software for the digital analysis of microscopic images.

### 2.7. Quantification Method

Quantification was performed on the same sections for both staining methods, with the Weidner method. This technique means counting the contour of the stained blood vessel wall by immunohistochemical methods and finally obtaining the number of microvessels per square millimeter [11]. Briefly, this method begins by examining the entire section under a photon microscope at a low objective to identify several “hot spots” with the highest density of blood vessels, followed by counting them using a high-magnification objective (×400). Mast cells were counted in the same microscopic fields. Three hot-spot locations with increased densities of mast cells and vessels were chosen for each individual case, and the arithmetic mean of the three microscopic fields was calculated for a final result. 

Immunoreactive mast cells were classified according to their granulated or degranulated state. Vessel and mast cell counts were performed in three different areas in all sections: intraepithelial, subepithelial and distant from the epithelium and in the depth of the connective tissue infiltrated with inflammatory cells.

### 2.8. Statistical Analysis

Data were collected using Microsoft Excel and analyzed in RStudio version 4.3.1. Continuous data are presented as means ± standard deviations for variables with Gaussian distributions or as medians with interquartile ranges for non-Gaussian distributions. Categorical data are presented as percentages. Differences between groups were assessed using Student’s t-test or analysis of variance for Gaussian continuous data, the Mann–Whitney U test or Kruskal–Wallis test for non-Gaussian continuous data, and the chi-squared test for categorical data. Normality was assessed using the Shapiro–Wilk and Kolmogorov–Smirnov tests, and equality of variance was evaluated with Levene’s test. Spearman’s correlation coefficient was used to evaluate the strength of association between non-Gaussian continuous variables. A priori sample size calculation was performed to achieve at least 80% statistical power at a 95% confidence level. A *p*-value less than 0.05 was considered statistically significant.

## 3. Results

### 3.1. Morphologic Analysis

From the histopathological point of view, we observed the changes that appear on the apex area of the tooth, thus achieving a better understanding of the destructive chronic inflammatory process. The connective tissue was analyzed by investigating the inflammatory infiltrate, which is mainly formed by lymphocytes, plasma cells and macrophages. The presence of mast cells in the inflammatory infiltrate was demonstrated immunohistochemically. 

**Figure 1:**
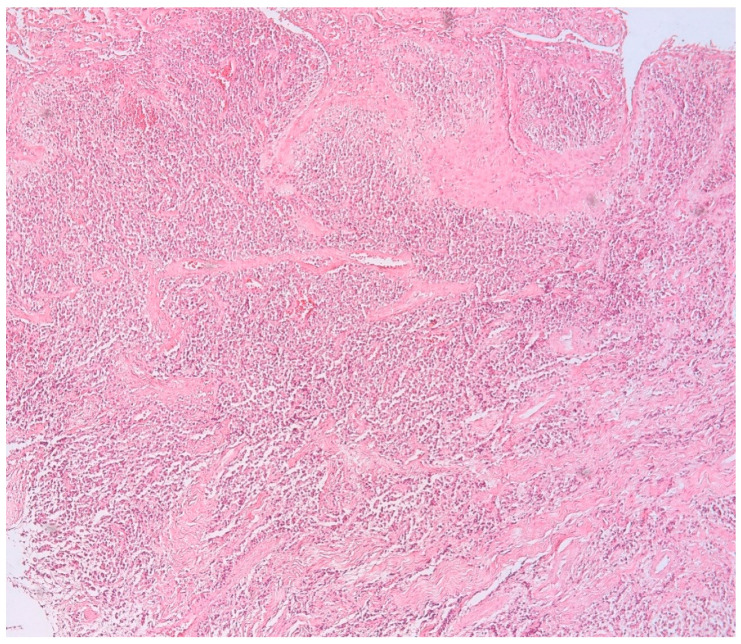
Connective apical granuloma, connective tissue with chronic inflammatory infiltrate, without epithelia, hematoxylin eosin staining, Ob.×100.

**Figure 2:**
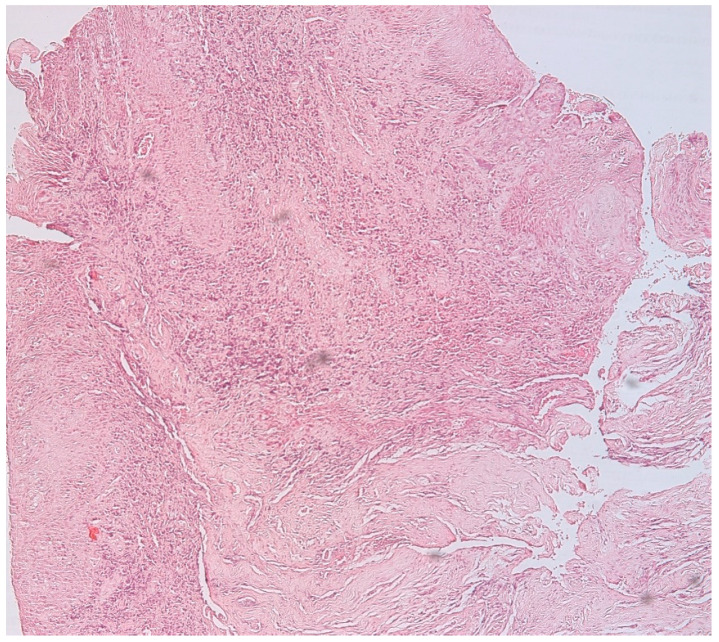
Epithelial apical granuloma, non-keratinized squamous stratified epithelia, inflammatory infiltrates, hematoxylin eosin staining, Ob.×100.

The presence of connective tissue with chronic inflammatory infiltrate, without epithelia, confirms the diagnosis of connective granuloma (*n* = 11) (Figure 1). In the cases included in our study, we also observed epithelial granulomas (*n* = 26) characterized by the presence of a non-keratinized stratified squamous epithelial tissue from the level of the oral cavity (Figure 2).

**Figure 3:**
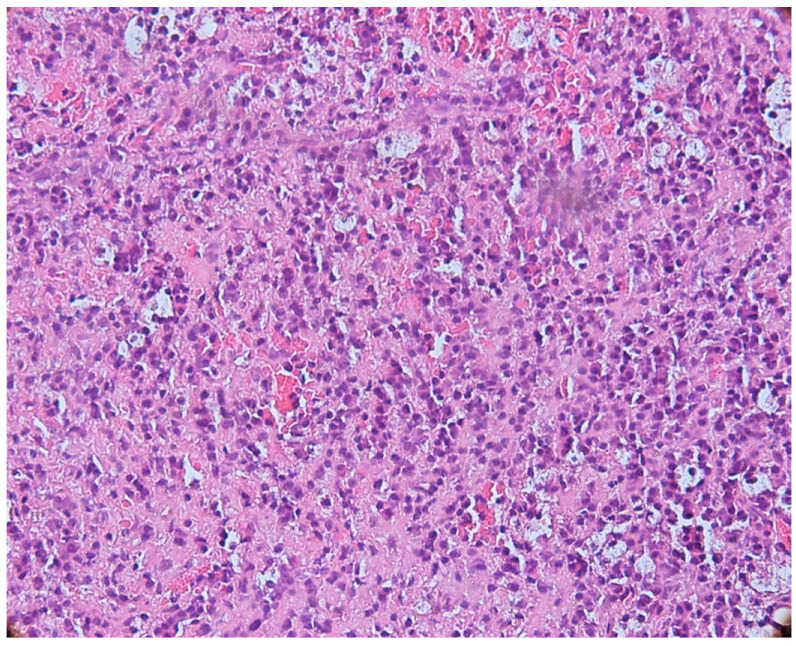
Periapical granuloma, inflammatory infiltrate with lymphocytes and plasma cells, hematoxylin eosin staining, ob.×200.

The microscopic appearance of apical granulomas was extremely varied from one case to another, most likely due to the etiopathogenic mechanisms that led to their formation, but also the reaction of the immune system specific to each patient. Most frequently, the histopathological aspect was dominated by the presence of inflammatory cells, represented, in particular, by lymphocytes and plasma cells, thus demonstrating the chronic aspect of the apical lesion (Figure 3).

**Figure 4:**
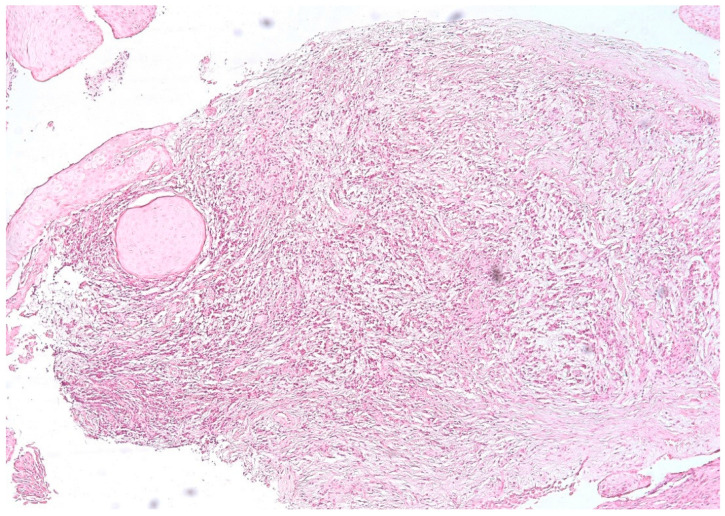
Periapical granuloma, peripheral zone with thick collagen fibers delimiting the granuloma from the rest of the periodontal connective tissue, hematoxylin eosin staining, ob.×100.

We identified an intense reaction of the connective matrix, with an increase in the density of collagen fibers, sometimes even with calcifications, which confirmed the long evolution of the granuloma. In all cases, we identified a higher density of connective tissue in the peripheral area of the lesions, which limited the granuloma from the rest of the periodontal connective tissue (Figure 4).

### 3.2. Mast Cells

**Figure 5:**
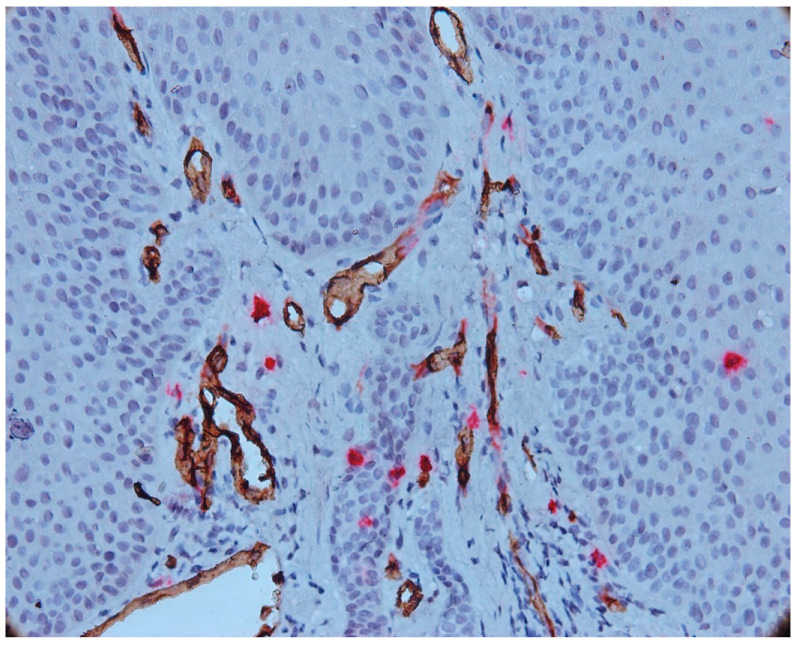
Intraepithelial mast cell, CD34-tryptase double immunostaining, Ob.×400.

With double immunostaining, we quantified the number of MCs and vessels in three different areas: intraepithelial, in the connective tissue near the epithelium (subepithelial) and in the depth of the connective tissue, in the area with inflammatory infiltrate. The immunohistochemical expression of tryptase in mast cells showed a cytoplasmic pattern, and immunoreactivity was observed in all cases of periapical granulomas (mast cells stained red). CD34-positive endothelial cells were stained brown (Figure 5). 

We noted the presence of intraepithelial mast cells, on average 1–4 mast cells/microscopic field in most cases of epithelial granuloma. Intraepithelial mast cells were small in size, round or oval in shape and not degranulated. In four cases included in our study, we noted more than four intraepithelial mast cells per microscopic field (7–9 MC/field). 

The number of intraepithelial mast cells was lower compared to the number of mast cells identified under the epithelium. Numerous vessels were observed to be subepithelial with a tendency to migrate towards the epithelium.

**Figure 6:**
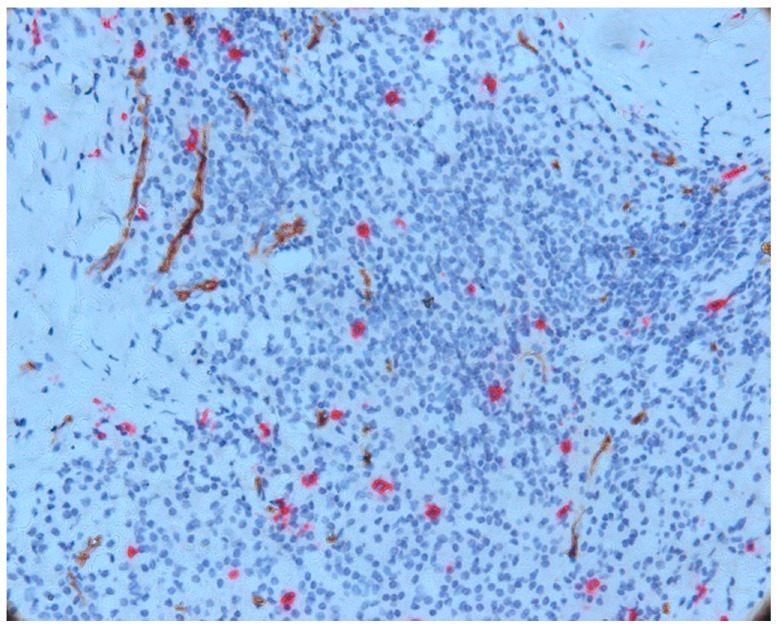
Mast cells and vessels in the area of the chronic inflammatory infiltrate, CD34-tryptase double immunostaining.

We also noticed an increased number of mast cells in the area of the inflammatory infiltrate, both in its central and peripheral areas. In most cases, an increased mast cell density (MCD) was observed in this area compared to the MCD seen in the intraepithelial and subepithelial sections. An increase in vascular density was also noted (Figure 6). 

Mast cells in the central part of the inflammatory infiltrate were small in size and most did not degranulate. In this area, we noticed only a few partially degranulated mast cells.

The mast cell density was calculated in the intraepithelial and subepithelial areas and in the connective tissue. The results are presented in Table 4.

Our results show an increase in mast cell density in periapical granulomas with an increased number of subepithelial mast cells compared to intraepithelial mast cells. The mast cells’ distribution was predominant among inflammatory cells, both at the periphery of the inflammatory infiltrate area and centrally.

### 3.3. Aspects of Microvessels

**Figure 7:**
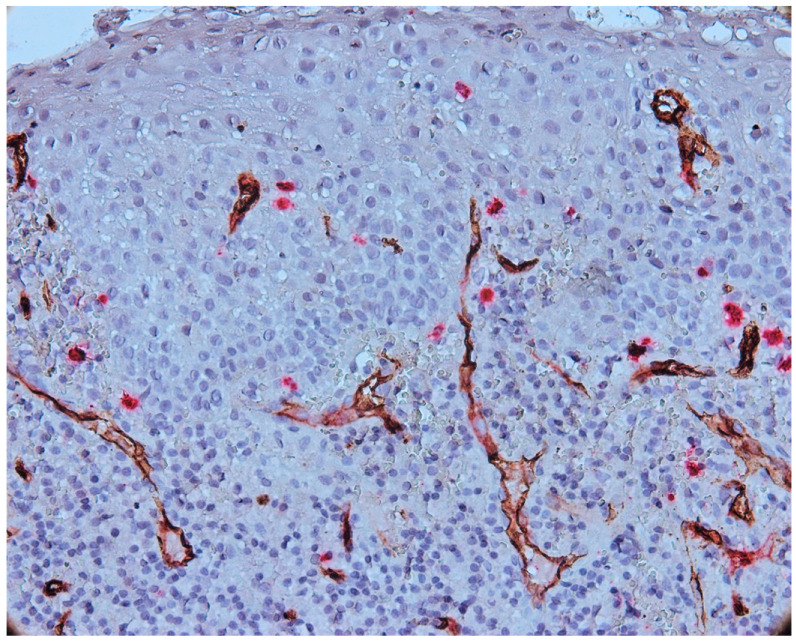
Vessels present in epithelium and subepithelial cells, CD34-tryptase double immunostaining, Ob.×400.

The intraepithelial vessels were small in size, with some showing a lumen, and some not presenting the lumen, as presented in Figure 7.

**Figure 8:**
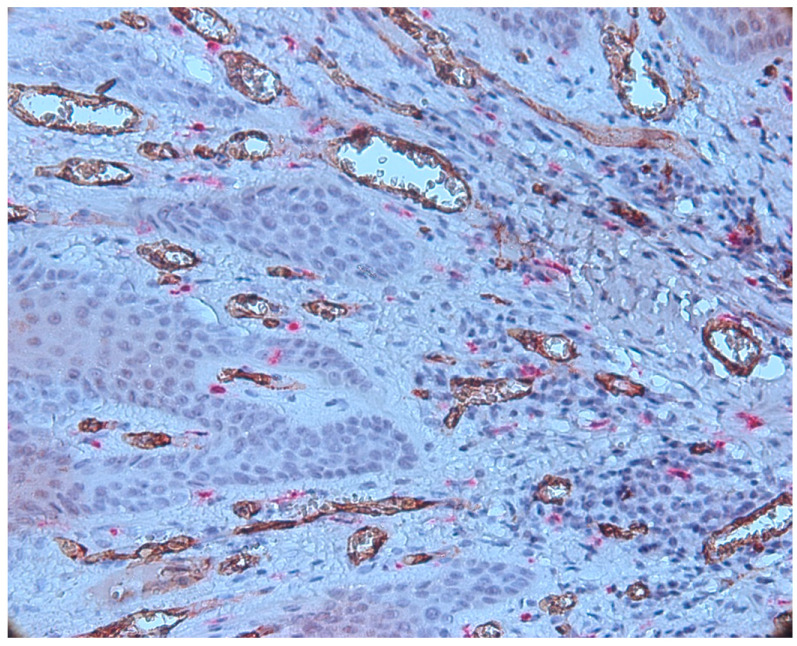
Numerous CD34-positive subepithelial vessels, CD34-tryptase double immunostaining, ob.×400.

Numerous vessels were observed in subepithelial layer with a tendency to migrate towards the epithelium. The blood vessels present in subepithelial cells were small to medium in size, some with a distinct lumen and others without a lumen (Figure 8).

**Figure 9:**
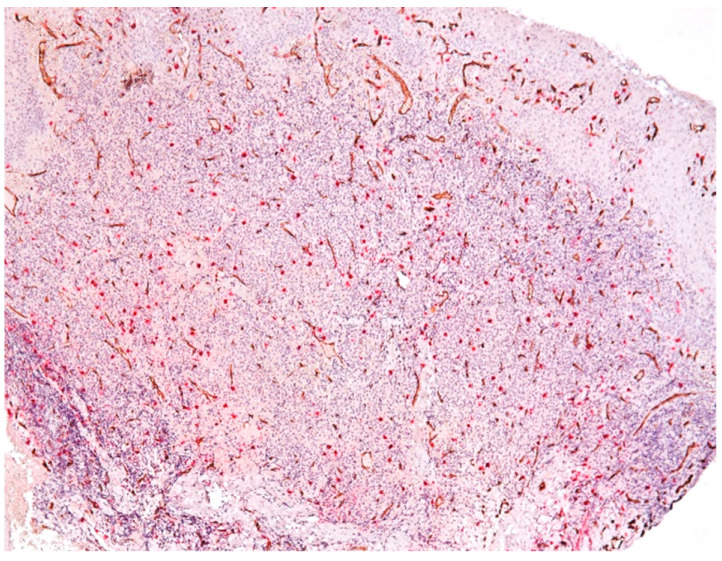
Vessels in the area of the chronic inflammatory infiltrate, CD34-tryptase double immunostaining.

The vessels present in the area of the inflammatory infiltrate were heterogeneous in morphology and size. In the central area of the inflammatory infiltrate, most of the vessels were small in size and did not have a lumen (Figure 9).

**Figure 10:**
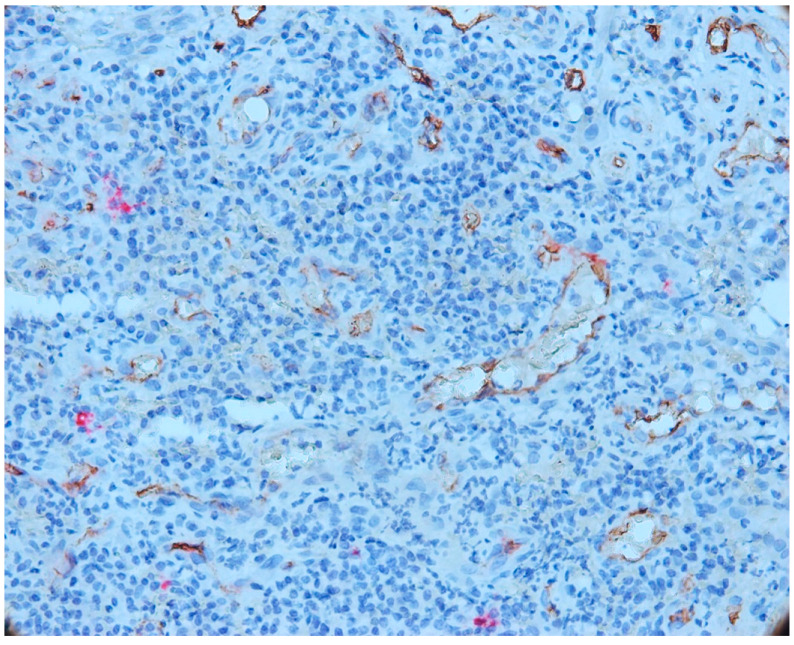
Vessels that presented a wide lumen, with protrusions towards the lumen of the endothelium, which signals the phenomenon of intussusception, CD34-tryptase double immunostaining, ob.×400.

We also observed some vessels that presented a wide lumen, with protrusions towards the lumen of the endothelium, which signals the phenomenon of intussusception (Figure 10).

**Figure 11:**
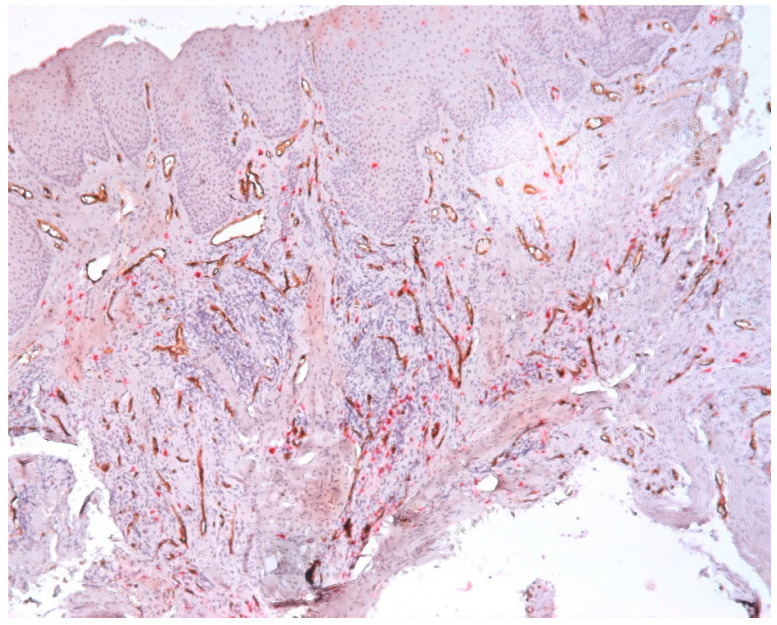
Vessels with variable morphology, CD34-tryptase double immunostaining, ob.×100.

We found great variability regarding the morphology of the vessels, namely the variable sizes; the irregular outline; the wide, narrow lumen; or the absence of the lumen and the ramification aspect. Large vessels with wide lumen were observed in the fibroconjunctive stroma. Vessels without a lumen and with a ramification aspect were observed in the area of the inflammatory infiltrate (Figure 11).

The microvessel density was calculated in the intraepithelial and subepithelial sections and in the connective tissue. The results are presented in Table 5.

Our results demonstrate an increased MVD and the presence of CD34-positive vessels with varied morphology (vessels of different sizes with or without lumen), the presence of intraepithelial vessels and the tendency of the vessels to migrate towards the epithelium. Predominantly, the small vessels without lumens were presented in the central area of the infiltrate. We also observed the presence of irregular vessels, which formed compartments in the area of the inflammatory infiltrate. Another interesting aspect was the vessels with a wide lumen, with protrusions towards the lumen of the endothelium, which has the phenomenon of intussusception.

### 3.4. MCD and MVD

The correlation matrix showed that there were significant positive correlations between intraepithelial mast cells and sub-epithelial mast cells (r = 0.6776, *p* < 0.001), subepithelial mast cells and subepithelial vessels (r = 0.5860, *p* < 0.001), and subepithelial vessels and connective tissue vessels (r = 0.5782, *p* < 0.001). There was also a significant negative correlation between connective tissue mast cells and subepithelial vessels (r = −0.1779, *p* = 0.292).

**Figure 12:**
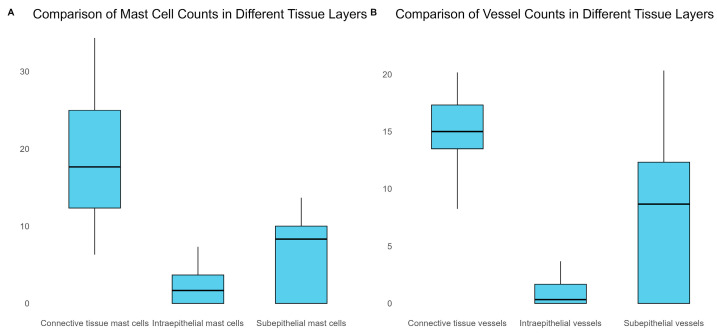
Comparison of MCD and MVD across various tissue layers.

The mean numbers of mast cells and vessels were lowest in the intraepithelial region and highest in the connective tissue. The connective tissue had the highest average numbers of both mast cells (18.7) and vessels (14.9) overall, as shown in Figure 12.

## 4. Discussion

An untreated endodontic infection is followed by a condition known as apical periodontitis, which develops as the body’s defensive mechanism against a microbial threat originating from the root canal system. Various histopathological categories of apical periodontitis, also known as periapical lesions, are eventually formed as a result of this dynamic interaction between microbial factors and host defenses. This complex process takes place at the interface between the infected radicular pulp and periodontal ligament and clinically appears as local inflammation, the resorption of hard tissues and the destruction of other periapical tissues [12]. 

Angiogenesis is a multilevel process that forms new blood vessels form existing ones. It can appear in both physiologic and pathologic conditions as periapical granuloma formation. It is one of the most well-known stromal factors contributing to the development of tumors such as periapical granulomas [13]. In our study, we have demonstrated the heterogeneity of the microvessels that appeared in various forms, with the presence or absence of the lumen, in an increased number of intraepithelial or subepithelial cells but with the high values located deep in the connective tissue.

Increased inflammation and angiogenesis are mechanisms that are present at the level of alveolar bone where periapical granulomas are present. In the scientific literature, the research is focused on the expression of the protein cluster differentiation 34 (CD34) and microvessel density (MVD) in malignant tissues generated from oral and odontogenic epithelia. The aim is to assess the angiogenesis process and various neo-angiogenesis levels [13,14]. Other studies assess the MVD in inflammatory diseases such as gingivitis, inflammatory periapical granulomas or cysts [2,6,15,16,17]. Our study confirms the variability of the microvessels and the fact that the connective tissue had the highest average numbers of vessels. 

A 120 kDa cell surface transmembrane phosphoglycoprotein called CD34 serves as an adhesion molecule between cells (1q32.2), and it is used for the quantification of both MVD and MCD [18]. Based on immunohistochemistry (IHC) techniques used in a range of tissue substrates, including cysts and neoplastic lesions of oral cavity, CD34 is a sensitive and specific marker, with this being the reason that our study used this technique [18,19]. 

Mast cells are present in periapical granulomas, suggesting their roles in the inflammatory mechanisms of these lesions. However, when under excessive stress, the mast cell may degranulate and release its active compounds, which support the inflammatory response [20,21]. The mast cells’ presence also plays a crucial role in periapical granulomas due to their implication in the initiation and promotion of angiogenesis, by secreting several potent angiogenic factors, such as heparin, histamine, VEGF and tryptase [22,23]. Nevertheless, the identification of mast cells in the present research in close proximity to blood vessels suggest that mast cells may play a role in angiogenesis in periapical granulomas. Our findings are confirmed by other published studies that confirm the crucial role played by the mast cells in the initiation, development and progression of the periapical granulomas, by being linked to increased vascular permeability, angiogenic response, collagen synthesis, the regulation of inflammation, bone resorption and extracellular matrix destruction [10,24]. 

The limitations of the study include the small size of the studied sample. We consider that our study can be performed on a large cohort of patients with periapical granulomas.

## 5. Conclusions

Our observations related to the presence of increased MCD in periapical granulomas suggest the role of these cells in the regulation of cellular immune mechanisms occurring in periapical lesions, probably balancing the alterative and reparative processes in the periapical tissue. The increased MVD and the variability of the microvessels found in the periapical granulomas shows a direct connection between the MCD and MVD. We demonstrate that MCD and MVD were lowest in intraepithelial cells and highest in the connective tissue. 

Although they are not directly involved in the body’s defense mechanisms, mast cells, through their mediators, contribute to the accumulation of an increased number of inflammatory cells. However, in the scientific medical literature, not many studies are present on mediators released by mast cells, and therefore, further studies on these cells are needed to define the role of mast cells in apical inflammatory lesions much more clearly. We also believe that tryptase-positive mast cells play an important role in angiogenic activity in periapical granulomas. Furthermore, because of the association between MC and vessels, other possible roles of tryptase should be considered in addition to angiogenic properties.

## Figures and Tables

**Table 1:** Profile of patients: age, gender and smoking habits.

Age	Gender	Smoking Status	Daily Cigarette Consumption
Mean (SD): 43.2 (11.9)	F: 17 (45.9%)	Non-smoker: 27 (73.0%)	Mean (SD): 17.7 (3.9)
Range: 24.0–72.0	M: 20 (54.1%)	Smoker: 10 (27.0%)	Range: 10.0–20.0

**Table 2:** Number of teeth extracted.

Extracted Tooth	
one	31 (83.8%)
two	5 (13.5%)
three	1 (2.7%)

**Table 3:** Localization of extracted teeth.

Extracted Tooth	
maxilla	23 (62.1%)
mandible	14 (37.9%)

**Table 4:** MCD distribution.

Intraepithelial Mast Cells	Subepithelial Mast Cells	Connective Tissue Mast Cells
Mean (SD): 2.1 (1.9)	Mean (SD): 6.5 (4.8)	Mean (SD): 18.7 (7.7)
Range: 0.0–7.3	Range: 0.0–13.7	Range: 6.3–34.3

**Table 5:** MVD distribution.

Intraepithelial Vessels	Subepithelial Vessels	Connective Tissue Vessels
Mean (SD): 1.4 (2.2)	Mean (SD): 7.8 (6.5)	Mean (SD): 14.9 (3.9)
Range: 0.0–9.7	Range: 0.0–20.3	Range: 0.0–23.2

## Data Availability

The data presented in this study are available on request from the corresponding author. The data are not publicly available due to restrictions regarding the privacy of the funding protocol.
